# Construction and high-throughput phenotypic screening of *Zymoseptoria tritici* over-expression strains

**DOI:** 10.1016/j.fgb.2015.04.013

**Published:** 2015-06

**Authors:** T.C. Cairns, Y.S. Sidhu, Y.K. Chaudhari, N.J. Talbot, D.J. Studholme, K. Haynes

**Affiliations:** Biosciences, University of Exeter, Stocker Road, Exeter, EX4 4QD, UK

**Keywords:** *Zymoseptoria tritici*, Functional genomics, Over-expression, High-throughput screen

## Abstract

Targeted gene deletion has been instrumental in elucidating many aspects of *Zymoseptoria tritici* pathogenicity. Gene over-expression is a complementary approach that is amenable to rapid strain construction and high-throughput screening, which has not been exploited to analyze *Z. tritici*, largely due to a lack of available techniques. Here we exploit the Gateway® cloning technology for rapid construction of over-expression vectors and improved homologous integration efficiency of a *Z. tritici* Δ*ku70* strain to build a pilot over-expression library encompassing 32 genes encoding putative DNA binding proteins, GTPases or kinases. We developed a protocol using a Rotor-HDA robot for rapid and reproducible cell pinning for high-throughput *in vitro* screening. This screen identified an over-expression strain that demonstrated a marked reduction in hyphal production relative to the isogenic progenitor. This study provides a protocol for rapid generation of *Z. tritici* over-expression libraries and a technique for functional genomic screening in this important pathogen.

## Highlights

• We generate a pilot library of 32 *Z. tritici* over-expression strains.
• We develop a high throughput technique for *in vitro* phenotypic screening.
• This protocol can probe both hyphal and budding *Z. tritici* morphologies.
• A putative transcription factor impedes hyphal biosynthesis when over-expressed.
• Genome wide functional analysis will enable discovery of novel virulence attributes.

## 1 Introduction

Gene disruption or deletion has been an essential technique for understanding the molecular basis of *Zymoseptoria tritici* virulence, including the role of MAP kinase signaling (Cousin et al., 2006; Mehrabi et al., 2006), transcription factor regulation (Kramer et al., 2009; Mirzadi Gohari et al., 2014), transport (Stergiopoulos et al., 2003) and effector biosynthesis (Marshall et al., 2011). In the rice blast fungus *Magnaporthe oryzae*, deletion of every gene encoding the autophagic apparatus clearly demonstrates that gene inactivation is a strategy amenable to functional genomics of plant pathogens (Kershaw and Talbot, 2009), and large-scale gene knock-out libraries have proven invaluable for elucidation of function in model and pathogenic fungi (Goncalves et al., 2011; Schwarzmuller et al., 2014). However gene deletion approaches have certain limitations. For example, essential genes cannot be functionally characterized by deletion. Additionally, for processes where multiple genes of similar function act synergistically, functional redundancy may make time-consuming characterization of null isolates ineffective. Moreover, phenotypic variation of nulls relative to wild-type isolates may be difficult to identify if the gene is not transcribed in standard laboratory culture, or transiently expressed at cryptic stages during infection assays.

Gene over-expression is a complementary approach to gene disruption or deletion. For infectious diseases of plants, researchers have utilized powerful heterologous expression technology facilitated by the availability of genetically tractable vectors and/or host systems. For example, a library of *Cladosporium fulvum* cDNAs was expressed in *Agrobacterium tumefaciens*, and four hypersensitive response-inducing genes were identified following leaf inoculation (Takken et al., 2000). Similarly, *Agrobacterium* mediated over-expression of *Phytophthora infestans* effectors *in planta* has revealed numerous responses by the host, including disease resistant hypersensitivity (Oh et al., 2009; Vleeshouwers et al., 2008). In addition to studies that probe the host/pathogen interface, heterologous expression has been implemented in model organisms, which is exemplified by work defining the *Z. tritici CYP51* gene in *Saccharomyces cerevisiae* (Cools et al., 2010).

In addition, over-expression of genes within the native organism has been used to elucidate basic biology for both model microorganisms and pathogens, especially when combined with high-throughput library construction and screening. Jin and colleagues used 2043 over-expression isolates in conjunction with 3627 transposon insertion mutants to identify the role of mitochondrial function in pseudohyphal growth of *S. cerevisiae* (Jin et al., 2008). In the pathogen *Candida albicans*, screening of a 257 open reading frame over-expression library identified a novel role in morphogenesis for 11 genes (Chauvel et al., 2012).

The aim of this study was to provide proof of principle for generating a genome-wide *Z. tritici* over-expression library and to develop a high-throughput technique for rapid functional screening *in vitro*. Accordingly, genes enriched amongst putative DNA binding proteins, kinases or GTPases were PCR amplified and cloned into pDONR207 to generate Gateway®Entry plasmids. Genes were subsequently shuttled into the newly described Gateway®Destination vector pYSKH3 (Sidhu et al., 2015). These *A. tumefaciens* adapted vectors were used to place genes under control of the *Z. tritici* translation elongation factor (*tef1*, Mycgr3G92705) promoter at the disrupted *Δku70* locus in strain HLS1000. The pilot library containing 32 *Z. tritici* over-expression strains was used to optimize a high-throughput screening protocol, where *in vitro* grown cells were rapidly and reproducibly pinned, using a Singer Rotor-HDA robot, onto solid agar containing various abiotic stressors that mimic stresses that might be encountered in the host. Using this approach, we identified an isolate that produced markedly less hyphae relative to the isogenic progenitor strain at the colony periphery under several stress conditions. This study provides a robust protocol for rapid generation of *Z. tritici* over-expression libraries and for high-throughput functional genomic screening. We also demonstrate proof of principle that this genome wide functional analysis will enable discovery of novel infection related biology.

## 2 Methods

### 2.1 Growth media

*Z. tritici* synthetic complete (ZTSC), a defined rich growth medium, comprising 6.9 g/l yeast nitrogen base without amino acids (ForMedium, UK), 0.79 g/l complete supplement mix (ForMedium), 20 g/l glucose, 20 g/l bacteriological agar (Lab, UK). Czapex Dox, a defined nutrient limiting medium, comprising 33.4 g/l Czapex Dox (Oxoid, UK), 20 g/l bacteriological agar (Lab).

Bacteria were grown in LB medium (Formedium) supplemented with kanamycin salt at 50 μg/ml (Sigma, UK) or gentamicin at 50 μg/ml (Sigma) where appropriate.

All strains were routinely stored at −80 °C in 50% (v/v) glycerol.

### 2.2 Plasmids used in this study

All plasmids were stored at −20 °C prior to use. Gateway®Entry vectors were constructed using pDONR207 (Invitrogen, UK) which contains a gentamicin resistance gene for selection in *Escherichia coli*. This plasmid also contains a *ccd*B gene flanked by *att*P sequences for Gateway® mediated recombination. For construction of over-expression vectors, we used pYSKH3 (Sidhu et al., 2015). This vector confers kanamycin resistance for selection in *E. coli* and *A. tumefaciens*. pYSKH3 contains a *ccd*B gene (co-ordinates 14,856–16,558) flanked with *att*R sites for Gateway® mediated recombination and selection. With regards to gene over-expression in *Z. tritici*, the Gateway® *ccd*B gene is flanked by an upstream sequence encoding (5′-3′): 26 bp t-border repeat (8733–8758), 1000 bp homology region to 5′UTR of the *Z. tritici Ku70* locus (8854–9853), 2361 bp functional *Ku70* gene (9854–12,214), 1382 bp of hygromycin resistance cassette (12,243–13,625) and 1200 bp translation elongation factor promoter (13,656–14,855). The 3′ region of the *ccd*B gene is flanked by a stop codon, 212 bp *CYC1* terminator (16,634–16,873), 800 bp region of 3′ UTR of *Ku70* locus (16,980–17,779) and a 26 bp t-border repeat (18,094–18,119, see Fig. 1A). Derivatives of pYSKH3 in which the *ccd*B gene was replaced with a DNA sequence encoding a *Z. tritici* gene were named pCCKH and numbered 1-32 (Supplementary Table S1).

### 2.3 Strains used in this study

*Z. tritici* strain HLS1000 (Sidhu et al., 2015) was used throughout. In this IPO323 derivative, the *Ku70* gene (Mycgr3G85040) of *Z. tritici* has been replaced with a G418 cassette using *A. tumefaciens* mediated transformation.

*E. coli* One Shot® *ccd*B Survival™ 2 T1^R^ was used for propagation of pDONR207 (Invitrogen, UK) and pYSKH3 (Sidhu et al., 2015). All Gateway®Entry and modified destination vectors were propagated in DH5α (Invitrogen, UK).

The kanamycin sensitive *A. tumefaciens* strain EHA105 (Hood et al., 1993) was used for *Z. tritici* transformation.

### 2.4 Construction of Gateway Entry vectors

For PCR amplification of each gene of interest, forward primers were designed to include the *att*B1 site (ggggacaagtttgtacaaaaaagcaggcttg and the first 20 bp of the gene, and reverse primers to include the *att*B2 site (ggggaccactttgtacaagaaagctgggtc) and the last 20 bp of the gene, excluding the stop codon. Predicted gene models were derived from the JGI (Goodwin et al., 2011). Primers were synthesised by Sigma (Table S1). PCR was conducted using Phusion® High-Fidelity DNA Polymerase (NEB) with a 65 °C primer annealing temperature and an extension of 0.5 min/kb, using *Z. tritici* IP0323 genomic DNA as template. PCR amplicons of predicted sizes were confirmed by gel electrophoresis, PEG purified, suspended in 10 μl TE buffer (40 mM TRIS base, 20 mM glacial acetic acid, 0.1 mM EDTA, pH8). For construction of Gateway®Entry vectors, 150 ng of pDONR207 was mixed with 2.5 μl of purified PCR product, 1 μl of Gateway® BP Clonase™ with TE buffer added to a total volume of 10 μl. Reactions were incubated at 25 °C for 24 h then treated with Proteinase K (Invitrogen, UK) following the manufacturer’s instructions. *E*. *coli* strain DH5α was transformed with 5 μl of each reaction mixture. Transformants were selected on LB supplemented with gentamicin (50 μg/ml). Colonies were grown over-night in LB medium with selection, and plasmids extracted using Plasmid Mini Kit (Qiagen, UK). In order to confirm recombination of the *ccd*B gene with the gene of interest, plasmids were digested with *Bsr*GI (NEB, UK), and digest reactions analyzed by gel electrophoresis. Putative Gateway®Entry vectors were Sanger Sequenced (Eurofins, UK) using primer GOXF (tcgcgttaacgctagcatgga).

### 2.5 Generation of *Z. tritici* over-expression plasmids

For construction of over-expression vectors pCCKH 1-32, 200 ng of *Z. tritici* Gateway®Entry plasmids were mixed with 200 ng of pYSKH3, 1 μl of LR Clonase™ with TE buffer added to a total volume of 10 μl. Reactions were incubated at 25 °C for 24 h then treated with Proteinase K (Invitrogen) following the manufacturer’s instructions. Reaction mixtures were transformed into *E*. *coli* strain DH5α as described above except transformants were selected on LB medium supplemented with kanamycin (50 μg/ml). Following plasmid extraction, plasmids were restriction mapped using either *Eco*RV, *Eco*RI or *Bam*HI (NEB). Confirmed over-expression vectors were named pCCKH 1-32. *Z. tritici* transformation with pCCKH plasmids 1-32 was conducted as described (Sidhu et al., 2015).

### 2.6 PCR confirmation of *Z. tritici* over-expression isolates

Genomic DNA was isolated from each putative transformant and integration of the over-expression construct at the *Δku70*::G418 locus determined by two PCR reactions (Figs. 2A and 2B). Firstly, forward primer Term_F (gctcgaaggctttaatttg) and reverse primer Ku70_EXT_R (ctggacatcaagcttcggat) yield a 1.8 kb product which is restricted to gDNA templates where the *Δku70*::G418 locus has been replaced with a pCCKH over-expression cassette. Genomic DNA templates were further screened with forward primer Hyg_F (ctcttctggaggccgtggt) and Ku70_EXT_R. These primers span a 1.57 kb 5′ region of the cassette, followed by sequence encoding the protein of interest and finally a 2.11 kb 3′ region. Accordingly, the size of the PCR product varies for each over-expression strain (Fig. 1A). This second PCR was used as a final screen to verify the gene of interest was integrated at the *ku70* locus. Single integration events were confirmed for eight transformants by Southern blot analysis (see Supplementary Methods S2).

### 2.7 High throughput phenotypic screen

#### 2.7.1 Preparation of cells and target/source plates

Robotic screening consisted of multiple rounds of pinning from a 96 well liquid cell suspension (source plate) onto multiple solid agar (target) plates. For the source plate, the *Z*. *tritici* HLS1000 *Δku70* progenitor strain and pCCKH over-expression isolates were obtained from long term storage at −80 °C in 50% (v/v) glycerol and grown for 7 days at 18 °C on ZTSC medium. Budding cells were scraped from plates using a sterile loop and added to 500 μl water, and washed by centrifugation at 5000 RPM for 10 min. Cells were re-suspended in water and cell density adjusted to 5 × 10^5^ cells/ml. Then 150 μl aliquots were arrayed in duplicate wells of a 96 well plate. Target agar plates consisted of 42 mls of ZTSC medium in Plus Plates (Singer Instruments, UK). For stress conditions, media was cooled to 42 °C and supplemented with hydrogen peroxide (1 mM, 2 mM, 5 mM), calcofluor white (25 μg/ml, 50 μg/ml), congo red (200 μg/ml/400 μg/ml) or caspofungin acetate (2.5 μg/ml). Technical duplicates were conducted for each condition with triplicate biological repeats.

#### 2.7.2 Robotic pinning

Robotic pinning was conducted with a Rotor-HDA pinning robot (Singer Instruments) using manufacturer’s software. The source plate was arrayed onto target agar plates using Repad 96LW pins (Singer Instruments). For collection of cells from source plate, a speed of 19 mm/s and plate retraction of 0.5 mm was used. Mixing of cell suspension between pinning was 3 rotations of 1.3 mm diameter at a speed of 25 mm/s. To ensure appropriate contact with the agar surface, the following parameters were used; pin pressure 22%, speed 15 mm/s, overshoot 1.9 mm, clearance 0.5 mm, diameter 1 mm. Using these parameters, a total of ∼1000 *Z. tritici* cells in 2 μl liquid suspension are pinned per sample.

#### 2.7.3 Imaging colony growth

Colony growth was observed daily. Images were captured using a M205FA (Leica, UK) stereo microscope at various magnifications using Leica application suite v 3.8.0. A V750 Pro Scanner (Epson, UK) was used for imaging gross plate layout.

### 2.8 Manual phenotypic screening

For manual reproduction of phenotypes detected by robotic pinning, cells were prepared as described above and serially diluted 1:10 in sterile water from densities 1 × 10^5^–1 × 10^1^ cells/ml. A total of 10 μl of each cell density was added to 25 ml agar plates containing concentrations of abiotic stressors indicated above.

For quantification of strain growth rates on defined nutrient limiting medium, cell density was adjusted to 200 cells/ml and 50 μl plated onto 25 ml Czapex Dox agar plates. Samples were incubated for 14 days at 18 °C, after which 50 colonies/strain were imaged using a Leica M205FA stereo microscope. Scale bars were added using Leica application suite v 3.8.0, images imported to Image J version 1.48v (NIH, Maryland, USA) and pixel:μM ratio calibrated. Colony area was measured by manually drawing around colony perimeter and recorded as mm^2^/colony. Triplicate biological replicates were conducted.

## 3 Results

### 3.1 Development of a pilot over-expression library in *Z. tritici*

**Supplementary Fig. S4:**
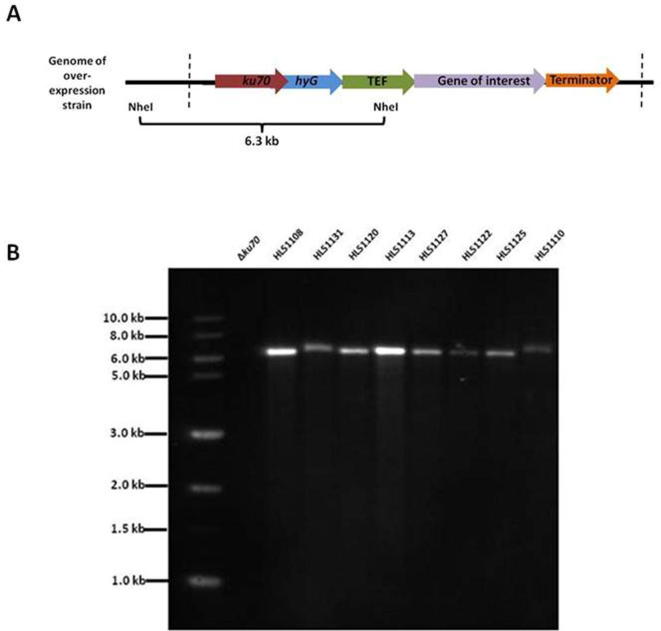
A: Schematic diagram of *ku70* locus replacement with pCCKH over-expression cassette. Dotted lines indicate the boundaries of homologous recombination between the cassette and recipient genome during transformation with pCCKH vectors. For Southern blot confirmation of single cassette integration, genomic DNA was digested with restriction endonuclease NheI, which generated a 6.3 kb fragment containing the hygromycin resistance gene (*hyG*). Southern blots were probed using a DIG labeled 1.1 kb fragment of the *hyG* gene. Image is not to scale. B: Confirmation of single cassette integration following transformation of strain HLS1000 with pCCKH. Southern blot analyses was conducted using progenitor strain and transformant gDNA which was digested with Nhe1. Blots were probed with a 1.1 kb bp fragment of the hygromycin cassette using the DIG labelling kit. Integration of the over-expression cassette at the *ku70* locus is indicated by a single Nhe1 fragment of 6.3 Kb observed for transformant gDNA. Southern blot was imaged using a G:Box (Syngene).

In order to construct over-expression vectors for *A. tumefaciens* mediated transformation of *Z. tritici*, we utilized the Gateway® experimental pipeline (see Sections 2.4 and 2.5). Forty genes were selected based on GO terms from JGI protein annotation (Supplementary Table S2). DNA sequences were PCR amplified with *att*B1/*att*B2 sites at the 5′ and 3′ flanks respectively, and recombined into donor plasmid pDONR207. All putative genes were successfully cloned using this approach, then subsequently recombined into Gateway® adapted destination vector pYSKH3 for *A. tumefaciens* mediated transformation of *Z. tritici*. In the pYSKH3 vector, genes are under 5′ control of the *tef1* promoter and followed immediately by a 3′ stop codon and terminator (Fig. 1A). Additionally, this vector has the following functionality in *Z. tritici*: (a) the over-expression cassette is targeted to the *Δku70*::G418 locus; (b) the *Ku70* gene (Mycgr3G85040) is reconstituted, and (c) the selection marker for transformation is hygromycin (Fig. 1B). Following *Z. tritici* transformation, hygromycin resistant isolates were recovered and PCR used to confirm integration of the over-expression cassette at the target locus (Fig. 1C). A total of 32 *Z. tritici* strains with *tef1* promoter directed over-expression of different genes at the *ku70* locus were generated using this methodology. Southern blot analyses (*n* = 8) confirmed single integration of the pCCKH cassette in recipient genomes (see Supplementary Fig. S4).

### 3.2 High throughput phenotypic screening of a *Z. tritici* over-expression library on oxidative and cell wall stress conditions

Given that *Z. tritici* infection is initiated on the leaf surface, we developed a high throughput assay for monitoring growth on solid substrates. This approach optimized a technique where isolates are robotically pinned onto agar plates +/− stress agents and growth compared. Stress conditions for library screening were selected in order to perturb *Z. tritici* processes which we hypothesize are required for infection. The fungal cell wall is a critical mediator of host-pathogen interactions, and extensive remodeling is required during germination and other differentiation events during pathogenesis. In order to determine genes for which *tef1* based expression at the *ku70* locus resulted in modulation of cell wall biosynthesis or architecture, isolates were screened on ZTSC agar plates supplemented with congo red or calcofluor white. These dyes perturb cell wall biosynthesis and integrity by binding chitin (Ram and Klis, 2006). Additionally, medium supplemented with caspofungin acetate was utilized to screen for genes which impact 1,3-beta-glucan synthase activity and/or antifungal resistance. Finally, we used hydrogen peroxide to probe for genes that respond to host derived oxidative stress (Shetty et al., 2007).

Strains were arrayed in technical duplicate onto solid agar using a Rotor-HDA pinning robot and colony morphology monitored for 16 days (Figs. 2A and 2B). The 32 over-expression isolates and *Δku70* control could be pinned onto target plates at approximately 4 plates/minute. Approximately 1000 cells per technical sample were pinned onto ZTSC agar and grown at 18 °C (see Section 2.7). A defined growth medium was used in order to avoid ambiguity with regards to nutritional content and to reduce variation between batches of media. Melanisation, which might impact sensitivity to numerous abiotic stressors, was not observed (Fig. 2B). Colony morphology on ZTSC plates reproducibly consisted of an area of mucoid budding cells surrounded by hyphae at the colony periphery (Figs. 2A and 2B). We therefore reasoned that this assay can be used to probe both budding and mycelial *Z. tritici* morphologies.

**Fig. 3:**
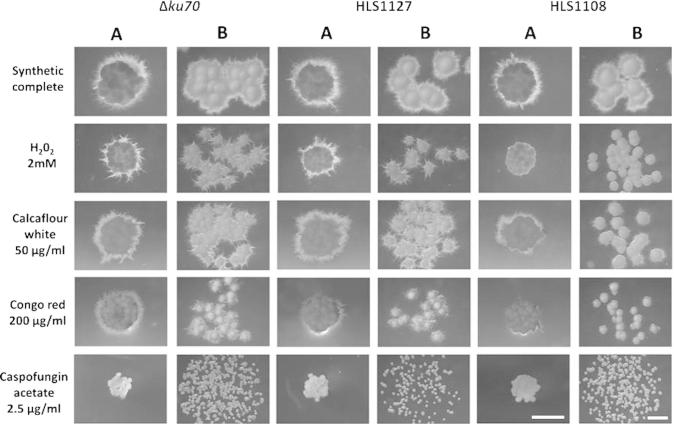
Phenotypes of *Z*. *tritici* strain HLS1108 detected by robotic pinning are reproducible manually. Microscopic images of colony morphology of *Z. tritici* HLS1000, the Δ*ku70* progenitor strain and two over-expression isolates following either robotic pinning (A) or serial dilution plating (B) on oxidative or cell wall stress conditions. Robotic pinned images and manual plated images were captured following 12 and 18 days growth at 18 °C respectively. Δ*ku70* and HLS1127 were used as isogenic progenitor and over-expression modified controls respectively. (A) For high throughput robotic screening, each strain was pinned in technical duplicate at a density of 5 × 10^5^. HLS1108 demonstrated reduced hyphal production at the colony periphery relative to control strains on medium supplemented with H_2_0_2_, calcofluor white and congo red. (B) In order to determine if this phenotype was cell density dependent, each strain was serially diluted and 10 μl grown on control or stress conditions at a density of 1 × 10^5^ to 1 × 10^1^ cells/ml. HLS1108 demonstrated marked reduction in hyphae at the colony periphery which was independent of cell density. All strains demonstrated comparable colony morphology when grown on medium supplemented with 2.5 μg/ml caspofungin acetate. Images were captured using a Leica M205FA stereo microscope. Scale bars in bottom right panels are 1 mm for either robotic or manually pinned images.

The progenitor and over-expression isolates demonstrated indistinguishable growth rates and colony morphology when grown on ZTSC medium (Figs. 2 and 3), indicating that homologous replacement of the *ku70* locus with pCCKH vectors does not detectably impact *in vitro* growth on defined rich medium. Furthermore, 31 over-expression isolates displayed comparable growth/morphology to the HLS1000 progenitor control on the various stress conditions tested, indicating *tef1* directed expression of these genes does not impact upon susceptibility to these compounds in this assay. However, the isolate HLS1108 displayed a striking reduction of hyphal growth at the colony periphery relative to progenitor control and other over-expression strains when perturbed with H_2_0_2_, congo red and calcofluor white (Figs. 2 and 3). The gene over-expressed in this isolate encodes a putative *Z. tritici* transcription factor (Mycgr3g111569, UniProt accession: F9XPA7). Interestingly, mucoid colony growth was not affected by stress conditions for this isolate. Indeed, under caspofungin acetate stress which completely inhibited hyphal growth, control and HLS1108 strains demonstrated comparable colony morphology (Fig. 3). We therefore hypothesize that (a) *tef1* directed expression of gene Mycgr3g111569 results in *Z. tritici* hyphal sensitivity to both oxidative and chitin mediated cell wall stressors; however, (b) budding growth is not detectably impacted by Mycgr3g111569 over-expression.

### 3.3 Manual confirmation of phenotypes identified by robotic screen

In order to confirm that phenotypes observed during high-throughput robotic screening were representative of standard *in vitro* experimentation, hyphal sensitivity of strain HLS1108 was investigated in simple manual assays. Budding cells were serially diluted to densities of 1 × 10^5^–1 × 10^1^/ml and pipetted onto agar containing ZTSC +/− stress conditions. Strains HLS1000 and HLS1127 were used as controls for isogenic progenitor strain and pCCKH modified isolates respectively. Following growth at 18 °C for 18 days, morphologies of all three strains were indistinguishable on ZTSC medium, but HLS1108 demonstrated reduced hyphal production on ZTSC medium + H_2_O_2_, congo red or calcofluor white relative to controls (Fig. 3b). We therefore consider the high throughput technique described above to be sufficiently similar to low throughput experimentation to allow robust hypothesis generation.

**Fig. 4:**
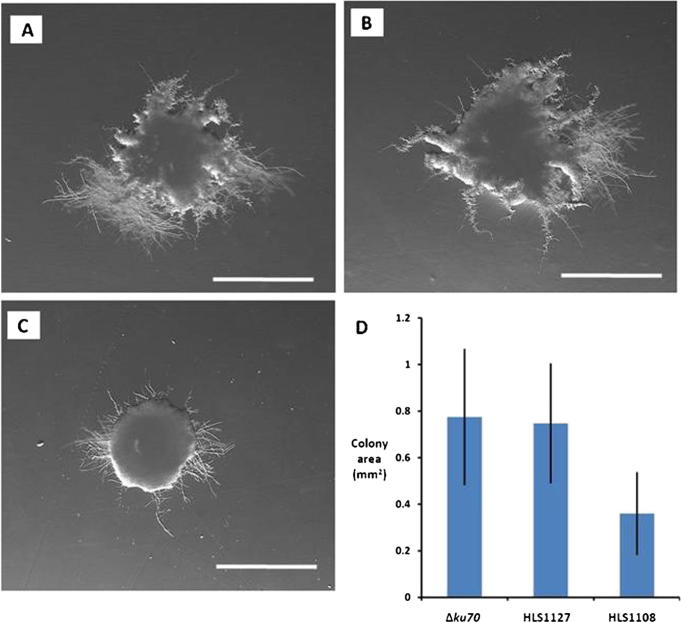
Quantitative variation of phenotypic difference between *Z. tritici* HLS1000 Δ*ku70*, HLS1127 and HLS1108. Characterization of strains following 14 days growth on Czapex Dox medium at 18 °C. Colonies were plated at a density of 10 CFU/25 ml plate. Growth on these conditions resulted in hyphal production in Δ*ku70* (A) and HLS127 (B), but was markedly reduced in HLS1108 (C). Triplicate biological replicates were conducted and area measured for a total of 150 CFU/strain (D). Differences in CFU area between HLS1108 and control strains were highly significant (Students *T*-test, *p* < 0.0001). Scale bars are 0.5 mm.

Given that over-expression strain HLS1108 demonstrated a hyphal sensitivity phenotype to cell wall and oxidative stressors, we reasoned that this gene may be antagonistic to *Z. tritici* hyphal growth. In order to test this hypothesis, Δ*ku70*, HLS1127 controls and HLS1108 budding cells were inoculated onto Czapex Dox medium at a density of 10 CFU per 25 ml plate (see Section 2.8). Czapex Dox medium promotes mycelial production by *Z. tritici* (Stergiopoulos et al., 2003). Colony area was markedly reduced in strain HLS1108 relative to control strains, which was highly statistically significant (Student’s *T*-test *p* < 0.0001, Fig. 4). No significant difference was observed between the progenitor and pCCKH modified controls. Microscopic examination revealed a reduction of hyphal production around the colony periphery in the over-expression isolate (Fig. 4). These data suggest that *tef1* driven over-expression of putative transcription factor Mycgr3g111569 impedes hyphal production of *Z. tritici*. We name this gene *alm*A, for *a lot less mycelium* A, and consider the over-expressing strain a useful tool for reverse engineering the molecular basis of hyphal growth in *Z. tritici*.

## 4 Discussion

The first transformation of *Z. tritici* was reported in 1998 (Payne et al., 1998), and a recent review by Orton and colleagues summarized the 23 genes that had been functionally characterized by 2011 (Orton et al., 2011). Despite revealing significant insights into *Z. tritici* biology, at this current rate of progress it will be approximately 50 years before 1% of the products of the 10,933 predicted *Z. tritici* genes have been functionally characterized. In order to improve the rate of functional characterization of *Z. tritici* genes, we provide proof of principle for generating a *Z. tritici* over-expression library and describe a high throughput technique for rapid phenotypic screening *in vitro*.

Construction of a *Z. tritici* over-expression library utilized the Gateway® cloning experimental pipeline for rapid vector construction, and a *ku70* null isolate, HLS1000, for improved homologous recombination during *Z. tritici* transformation. Using these tools we were able to successfully integrate 80% of genes at the *ku70* locus under control of the *tef1* promoter, which is a pass rate consistent with similar studies in other pathogenic fungi (Chauvel et al., 2012). The molecular tools used for library construction confer additional functionality to mitigate both false positive and false negative results that might confound interpretation of data from high throughput functional genomic screens. Firstly, targeting over-expression cassettes to the *ku70* locus is preferable to random integration, which might lead to disruption of other coding sequences, or occur at transcriptionally silent/and or dispensable regions of the *Z. tritici* genome. Additionally, while the *Z. tritici ku70* null mutant used in this study improves homologous targeting, genome instability in this strain may introduce phenotypic aberrations in derivative isolates. Genome instability is a well documented phenotype of *ku* strains, which is supported by sensitivity of the *Δku70* isolate to ultraviolet directed DNA damage (Sidhu et al., 2015). Accordingly, vector pYSKH3 contains the full coding sequence of the wild-type *Z. tritici Ku70* gene (Mycgr3G85040) to restore wild-type levels of genome stability.

Next, a high throughput *in vitro* screening protocol was optimized for a Rotor-HDA pinning Robot (Singer Instruments). Using this platform, 96 well cell suspensions can be arrayed on to four agar plates per minute. This assay is simple to scale up by using multiple 96 well source plates and/or increasing the number of conditions probed by target plates. Although beyond the scope of this study, we are optimizing *in vitro* conditions which probe other possible elements of *Z. tritici* niche adaptation during infection, such as macro and micro nutrient depletion, desiccation and osmotic fluctuations. While we have screened over-expression isolates, it would be easy to screen other *Z. tritici* libraries, such as naturally occurring wild-type strain collections or large numbers of gene knock-outs. Additionally, it will be simple to adopt the platform to liquid growth assays by changing target agar plates to 96 well liquid suspensions.

While functional characterization of genes by over-expression confers several advantages to gene disruption or deletion, such as facilitating characterization of essential genes and avoiding issues of functional redundancy, there are certain limitations. For example, feedback mechanisms required for proper transcription factor function during native expression might be distorted by significantly elevated protein levels. For kinases, over-expression can lead to inactive protein subcomplexes (Gibson et al., 2013). More specifically for this study, while *tef1* controlled over-expression of pYSKH3 derivatives in *Z. tritici* has recently been validated using a GFP reporter (Sidhu et al., 2015), as protein levels were not confirmed in this study we cannot rule out that post transcriptional silencing occurred for certain genes. Additionally, we assume that the *tef1* promoter is constitutively active, although the activity of this promoter might vary for certain stress conditions. Therefore, differences between the *tef1* promoter activities between experimental conditions might explain why strain HLS1108 maintained hyphal production at the colony periphery when grown on synthetic complete medium but not on Czapex Dox or stress conditions. Notwithstanding these limitations, the high throughput screen described in this study demonstrated over-expression of the putative *Z. tritici* transcription factor *alm*A results in hyphal sensitivity to oxidative and chitin based cell wall stress. A simple growth assay on hyphal inducing conditions demonstrated a reduction in hyphal growth in this strain relative to the progenitor strain. We are currently working to identify the downstream targets for this transcription factor, which might identify important attributes of *Z. tritici* hyphal growth or architecture.

## 5 Conclusion

This study has presented the construction of a library of *Z. tritici* over-expression strains and developed a high throughput *in vitro* screen. The identification of a mutant with reduced hyphal production provides proof of principle that these techniques will facilitate genome-wide functional genomics analysis of *Z. tritici* pathogenicity via over-expression screens, and reveal novel biological functions.

## Figures and Tables

**Fig. 1A:**
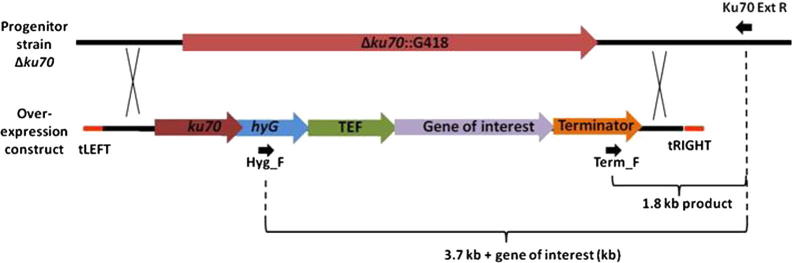
Schematic diagram of homologous recombination between Δ*ku70* progenitor strain and over-expression cassette at the *ku70*::G418 locus. Over-expression constructs were generated using plasmid YSKH3, placing genes of interest under control of the *Z. tritici tef1* promoter. The entire cassette is flanked by two 26 bp t-border repeats (tLEFT/tRIGHT) for *A. tumefaciens* mediated transformation. Homologous recombination (black crosses) occurs between cassette and recipient genome by 1.0 and 0.8 bp sequences of 5′ and 3′ UTR respectively. The cassette contains the entire 2.3 kb coding sequence of *Z. tritici Ku70* gene (Mycgr3G85040) directly downstream of the native UTR. A hygromycin resistance cassette (*hyG*, 1.3 kb) confers selection of putative transformants. Immediately downstream of the gene of interest is a 0.2 kb *CYC1* terminator. Hygromycin resistant transformants were isolated and integration of the cassette confirmed by two PCR reactions using primers Term_F/Ku70_Ext_R or Hyg_F/Ku70_Ext_R (black arrows).

**Fig. 1B:**
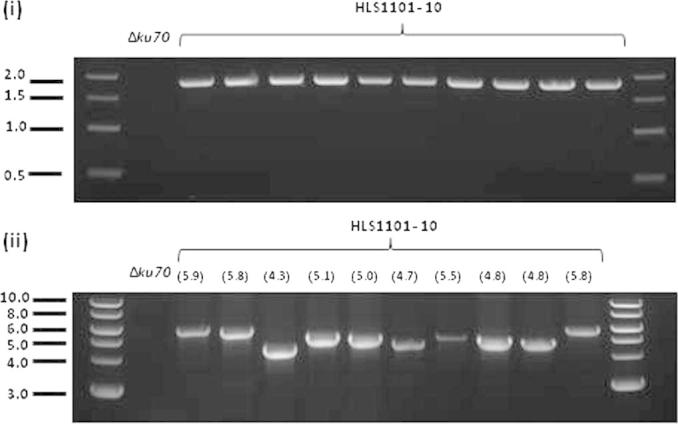
PCR confirmation of 10 over-expression cassette homologous recombination events at the Δ*ku70::G418* locus. Gel electrophoresis image of PCR using primers Term_F/Ku70_EXT_R (i) or Hyg_F/Ku70_EXT_R (ii) with either Δ*ku70* or putative transformant (HLS1101-HLS1110) genomic DNA as template. PCR using primers Term_F/Ku70_EXT_R displayed the predicted 1.8 kb product using transformant but not Δ*ku70* gDNA. PCR products from Hyg_F/Ku70_EXT_R produced bands of predicted sizes with genomic DNA isolated from transformants but no product from Δ*ku70* gDNA. Numbers in parenthesis indicate predicted size of PCR product in kb. Marker was 1 kb DNA Ladder (NEB).

**Fig. 1C:**
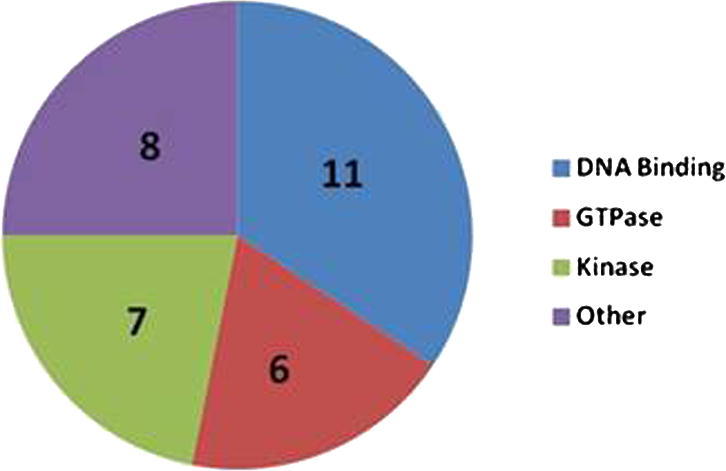
Distribution of PCR confirmed over-expression genes amongst putative functional cohorts. Genes were assigned function based on PFAM/GO predictions from the JGI.

**Fig. 2A:**
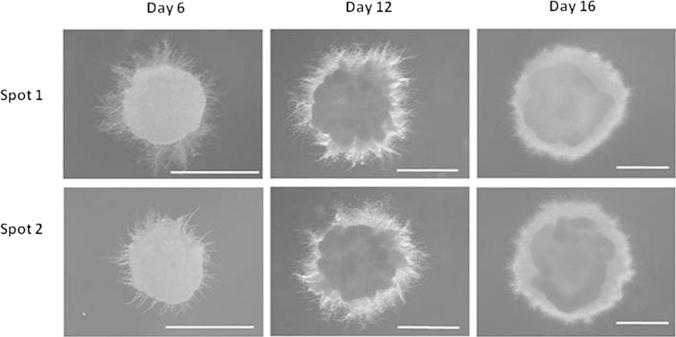
Colony growth following highthroughput robotic pinning demonstrates budding and hyphal morphologies of *Z. tritici*. Displayed are images of technically duplicated spots of the *Z*. *tritici* Δ*ku70* strain HLS1000 following 6, 12 and 16 days growth at 18° on ZTSC medium. Approximately 1000 cells in a volume of 2 μl were pinned onto agar. Colonies consisted of a budding mucoid morphology surrounded by hyphae at the periphery. Images were captured using a Leica M205FA stereo microscope. Scale bars are 1 mm length.

**Fig. 2B:**
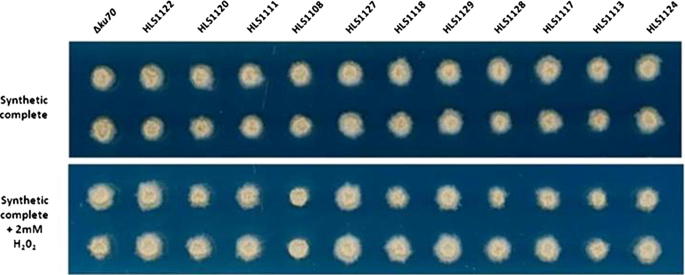
High throughput robotic phenotyping of over-expression strains. Representative image of colony morphology of the *Z*. *tritici* Δ*ku70* progenitor strain HLS1000 and 11 over expression strains following robotic pinning and growth at 18 °C for 16 days. Each strain was pinned in technical duplicate at a density of 1000 cells on either ZTSC medium or ZTSC supplemented with 2 mM H_2_0_2_. Colony morphology between control and stress conditions were identical at this time point for all strains except HLS1108, which demonstrated marked reduction in hyphal production at the colony periphery. Image was captured using an Epson V750 Pro Scanner.
