# Supplementary material for: Construction and high-throughput phenotypic screening of Zymoseptoria tritici over-expression strains
Source: Fungal Genet Biol. 2015 Jun;79:110–7. doi: 10.1016/j.fgb.2015.04.013 (PMC4502453; doi:10.1016/j.fgb.2015.04.013)
Supplement: Supplementary Table S1 [file mmc1.docx]

| **Gene over expressed** | **Forward primer** | **Reverse Primer** | **pDest Vector** | ***Z. tritici* over-expression isolate** |
| --- | --- | --- | --- | --- |
| Mycgr3G92404 | ggggacaagtttgtacaaaaaagcaggcttgatgATGATTCAGCCAACTCGAGT | ggggaccactttgtacaagaaagctgggtcCTCCCGTAACCTCTCAACAC | pCCKH01 | HLS1101 |
| Mycgr3G99365 | ggggacaagtttgtacaaaaaagcaggcttgatgCCTTGCTTCAAGGGCCTTGC | ggggaccactttgtacaagaaagctgggtcAAGCTCCTCATCTCCATCTT | pCCKH02 | HLS1102 |
| Mycgr3G64933 | ggggacaagtttgtacaaaaaagcaggcttgatgGAACAACTCCTCCTCCCTCA | ggggaccactttgtacaagaaagctgggtcAGATCCCTTCAACACCATGC | pCCKH03 | HLS1103 |
| Mycgr3G49843 | ggggacaagtttgtacaaaaaagcaggcttgatgTTTCGGGCAGCCAGCGTAAG | ggggaccactttgtacaagaaagctgggtcTGCAAGCCACTCATCCCGCA | pCCKH04 | HLS1104 |
| Mycgr3G92288 | ggggacaagtttgtacaaaaaagcaggcttgatgGCGCTCGTCGAATACTCCGA | ggggaccactttgtacaagaaagctgggtcACCCCACCCATATTTACTTG | pCCKH05 | HLS1105 |
| Mycgr3G104022 | ggggacaagtttgtacaaaaaagcaggcttgatgAGCGCCTACAGCAAGCAGAA | ggggaccactttgtacaagaaagctgggtcGGTCGCTGCGGAAAACTTGG | pCCKH06 | HLS1106 |
| Mycgr3G103568 | ggggacaagtttgtacaaaaaagcaggcttgatgGACGTTCTCTCCGCATCTTC | ggggaccactttgtacaagaaagctgggtcAGTAGTATCACCACCATGAC | pCCKH07 | HLS1107 |
| Mycgr3G111569 | ggggacaagtttgtacaaaaaagcaggcttgatgTCTCTCCACACCCCAGCACC | ggggaccactttgtacaagaaagctgggtcAGCATGGACCTCAGTATTCG | pCCKH08 | HLS1108 |
| Mycgr3G97382 | ggggacaagtttgtacaaaaaagcaggcttgatgGAGCACCGCGCCGTGAGCAA | ggggaccactttgtacaagaaagctgggtcCTTGGACGGCTTCTCGTTCT | pCCKH09 | HLS1109 |
| Mycgr3G94193 | ggggacaagtttgtacaaaaaagcaggcttgatgGCGACCGCGCCTTGCCCCAC | ggggaccactttgtacaagaaagctgggtcCTCCTCATCATCATCATCCA | pCCKH10 | HLS1110 |
| Mycgr3G46302 | ggggacaagtttgtacaaaaaagcaggcttgatgCTCTGCTGCTTGAAGCGCGC | ggggaccactttgtacaagaaagctgggtcTGAAGCAGATAAGTCCAGCC | pCCKH11 | HLS1111 |
| Mycgr3G94584 | ggggacaagtttgtacaaaaaagcaggcttgatgCCTACTATGCCCAATTTCGA | ggggaccactttgtacaagaaagctgggtcTTTGTCAAATCTACTCTTTT | pCCKH12 | HLS1112 |
| Mycgr3G33027 | ggggacaagtttgtacaaaaaagcaggcttgatgGCCTCTGTGTGCCTGGTCGG | ggggaccactttgtacaagaaagctgggtcAAGATTCCACTCCGTCCTGC | pCCKH13 | HLS1113 |
| Mycgr3G109078 | ggggacaagtttgtacaaaaaagcaggcttgatgTCCGCGTCTGACCAACAATC | ggggaccactttgtacaagaaagctgggtcTGTGTTCCTCAAAAACACCC | pCCKH14 | HLS1114 |
| Mycgr3G76263 | ggggacaagtttgtacaaaaaagcaggcttgatgCTCAAGGCGTGGCTGCGCGA | ggggaccactttgtacaagaaagctgggtcTCCTCGCCCATAAAGCCCAG | pCCKH15 | HLS1115 |
| Mycgr3G64085 | ggggacaagtttgtacaaaaaagcaggcttgatgGAAGAGCAAGTAGAACGATT | ggggaccactttgtacaagaaagctgggtcACCAGTCTCCTCCGCAACCA | pCCKH16 | HLS1116 |
| Mycgr3G111096 | ggggacaagtttgtacaaaaaagcaggcttgatgGATGAAGAGATGCAGTCGGG | ggggaccactttgtacaagaaagctgggtcCCCAGCACTACTCAACCAAG | pCCKH17 | HLS1117 |
| Mycgr3G51460 | ggggacaagtttgtacaaaaaagcaggcttgatgGGCGCCTGGGCACTTACGCT | ggggaccactttgtacaagaaagctgggtcATTCCCGCAATACCTTCGAT | pCCKH18 | HLS1118 |
| Mycgr3G102517 | ggggacaagtttgtacaaaaaagcaggcttgatgTTCGCCCGCACCCTCCGCCA | ggggaccactttgtacaagaaagctgggtcTGAGTTTCCACCGCTGCCAC | pCCKH19 | HLS1119 |
| Mycgr3G36302 | ggggacaagtttgtacaaaaaagcaggcttgatgCCAGCGAGCCGCACTGCGCG | ggggaccactttgtacaagaaagctgggtcCACAGCACCATAGAGATCCC | pCCKH20 | HLS1120 |
| Mycgr3G90147 | ggggacaagtttgtacaaaaaagcaggcttgatgGCATACGCCGACGCCAGATT | ggggaccactttgtacaagaaagctgggtcTGGTTCGCTTTTGTACGTGA | pCCKH21 | HLS1121 |
| Mycgr3G35076 | ggggacaagtttgtacaaaaaagcaggcttgatgGACGCTCCATCACAGCGGTC | ggggaccactttgtacaagaaagctgggtcCCAACTATCATTGTCAAACA | pCCKH22 | HLS1122 |
| Mycgr3G70110 | ggggacaagtttgtacaaaaaagcaggcttgatgCCGCCTCAGATCAAGCAAGA | ggggaccactttgtacaagaaagctgggtcCTCCCCAGCCATAGCAGCAT | pCCKH23 | HLS1123 |
| Mycgr3G111645 | ggggacaagtttgtacaaaaaagcaggcttgatgGACTACGACGATACCTCAGG | ggggaccactttgtacaagaaagctgggtcGTCGCTGGGCGTGGAAGATC | pCCKH24 | HLS1124 |
| Mycgr3G62811 | ggggacaagtttgtacaaaaaagcaggcttgatgGCGGCTTCGGCATCTGCATC | ggggaccactttgtacaagaaagctgggtcTCCCTCTCGAAATCCTGCCA | pCCKH25 | HLS1125 |
| Mycgr3G89777 | ggggacaagtttgtacaaaaaagcaggcttgatgGCAGATGTTGCGAACCGCGC | ggggaccactttgtacaagaaagctgggtcCTGCTCCTCAATGCGCGAAG | pCCKH26 | HLS1126 |
| Mycgr3G109829 | ggggacaagtttgtacaaaaaagcaggcttgatgGAGATCCCCAAGACCGAGGC | ggggaccactttgtacaagaaagctgggtcTCCCGCGAAAAATTGTAAGC | pCCKH27 | HLS1127 |
| Mycgr3G88104 | ggggacaagtttgtacaaaaaagcaggcttgatgACCTCCGCCGCCGCCGACGA | ggggaccactttgtacaagaaagctgggtcACCATGCTCGTCTTCACTCA | pCCKH28 | HLS1128 |
| Mycgr3G95055 | ggggacaagtttgtacaaaaaagcaggcttgatgCCGATCCCGCCTGGCCAGAA | ggggaccactttgtacaagaaagctgggtcCATAATCACACAACAAGAGC | pCCKH29 | HLS1129 |
| Mycgr3G104515 | ggggacaagtttgtacaaaaaagcaggcttgatgTCGAACCCTCGAGCCAAGCC | ggggaccactttgtacaagaaagctgggtcGATGCCCAACGGATTGACAG | pCCKH30 | HLS1130 |
| Mycgr3G91519 | ggggacaagtttgtacaaaaaagcaggcttgatgCAGCGATACCTTCAAGATCC | ggggaccactttgtacaagaaagctgggtcAGTCAAGCTCTTTCGCCTGC | pCCKH31 | HLS1131 |
| Mycgr3G68887 | ggggacaagtttgtacaaaaaagcaggcttgatgTCCTTCGCTCGGAATCTCCT | ggggaccactttgtacaagaaagctgggtcATCCAACAGACGGGTGATCA | pCCKH32 | HLS1132 |

**Table S1: List of genes over-expressed in *Z. tritici* in this study.** Forward and reverse primers for amplification of predicted gene of interest are shown in block capitals. Predicted gene models were derived from the JGI. attB sequences appended to forward or reverse primers for Gateway® recombination into pDONR207 are shown in lowercase. Destination plasmids (pDest) used for transformation of *Z. tritici* are derivatives of pYSKH3, which enables TEF controlled over-expression (Sidhu et al. this issue, and Figure 1A). In these plasmids, the *ccd*B gene of pYSKH3 was replaced with a DNA sequence encoding the *Z. tritici* gene of interest using the Gateway®LR reaction. Plasmids were named pCCKH01-32, and corresponding *Z. tritici* over-expression isolates named HLS1101 - HLS1132.
