# Supplementary material for: Construction and high-throughput phenotypic screening of Zymoseptoria tritici over-expression strains
Source: Fungal Genet Biol. 2015 Jun;79:110–7. doi: 10.1016/j.fgb.2015.04.013 (PMC4502453; doi:10.1016/j.fgb.2015.04.013)
Supplement: Supplementary Methods S2 [file mmc2.docx]

**Supplementary methods S2**

**S.2 Southern blot analyses of progenitor strain and transformant genomic DNA**

In order to confirm single cassette integration in the recipient genome, 8 transformant strains were tested and the progenitor strain HLS1000 used as an un-transformed control. Five μg of gDNA was digested for 8 hours at 37 °C with the restriction endonuclease NheI (NEB, UK, see Figure S1A). Digested reactions were separated on a 1% (w/v) agarose gel and then depurinated (250 mM HCl), denatured (1.5 M NaCl, 0.5 M NaOH), neutralised (1.5 M NaCl, 0.5 M Tris-HCl, pH 7.5,) and equilibrated in 20 x SSC (3 M NaCl, 300 mM sodium citrate, pH 7). DNA was transferred overnight onto a nylon membrane (Roche, UK) after which the DNA was fixed to the membrane using crosslink function on a Stratagene UV Stratalinker 1800. DIG Easy Hyb solution (Roche, UK) was pre-warmed to the probe hybridisation temperature (52 °C) and the membrane prehybridised in DIG Easy Hyb for 30 minutes. The DNA probe was amplified from template pCCYH1, using primers DIG_F (GGTGCACGATAACTTGGTG) and DIG_R (CTATTCCTTTGCCCTCGGA) and the PCR DIG Probe synthesis kit (Roche, UK). The probe spans a 1.1 kb region of a *trpC* promoter and *hygG* gene. Approximately 50 ng of probe was denatured for 10 minutes at 100 °C for 5 minutes, which was then added to 10 ml prewarmed DIG Easy Hyb solution. The membrane was incubated overnight at 52 °C, incubated with low stringency wash buffer (2 x SSC, 0.1% (w/v) SDS) at room temperature, followed by high stringency wash buffer (0.5 x SSC, 0.1% (w/v) SDS) at 65 °C, washed (0.1 M Malic acid, 0.15 M NaCl (pH 7.5), 0.3% (v/v) Tween 20) and blocked (1% (w/v) blocking solution (Roche, UK) in 0.1 M Malic acid, 0.15 M NaCl, pH 7.5) for 1 hour at room temperature. The antibody (Anti-Digoxigenin-AP Fab fragments, Roche) was used at a dilution of 1 in 10000 in 30 ml blocking solution for 30 minutes with gentle agitation. The membrane was then washed in wash buffer and incubated with detection buffer (0.1 M Tris-HCl, 0.1 M NaCl, pH 9.5) for 5 minutes. Southern blot was imaged using a G:Box (Syngene, UK) which demonstrated a single, labelled 6.3 kb DNA fragment in the transformant samples but not the progenitor isolate (Figure S1B).
